# Hypermethylation in Calca Promoter Inhibited ASC Osteogenic Differentiation in Rats with Type 2 Diabetic Mellitus

**DOI:** 10.1155/2020/5245294

**Published:** 2020-03-04

**Authors:** Lei Wang, Feng Ding, Shaojie Shi, Xingxing Wang, Sijia Zhang, Yingliang Song

**Affiliations:** State Key Laboratory of Military Stomatology & National Clinical Research Center for Oral Diseases & Shaanxi Engineering Research Center for Dental Materials and Advanced Manufacture, Department of Implantology, School of Stomatology, The Fourth Military Medical University, Xi'an, China

## Abstract

The abnormal environment of type 2 diabetes mellitus (T2DM) leads to a substantial decrease in osteogenic function of stem cells. However, the gene sequence does not vary before and after disease for the patient. This phenomenon may be related to changes in osteogenesis-related gene expression caused by DNA methylation. In this study, we established T2DM models to extract adipose-derived stem cells (ASCs) for different gene identifications through DNA methylation sequencing. Specific fragments of methylation changes in the target gene (Calca) were identified by IGV analysis. CGRP was applied to compare the effects on ASCs-T2DM morphology via phalloidin staining, proliferation through CCK-8 assay, and osteogenic differentiation with osteogenic staining, qPCR, and repair of calvarial defect. Furthermore, 5-azacytidine (5-az) was used to intervene ASCs-T2DM to verify the relationship between the methylation level of the target fragment and expression of Calca. We found that the DNA methylation level of target fragment of Calca in ASCs-T2DM was higher than that in ASCs-C. CGRP intervention showed that it did not change the morphology of ASCs-T2DM but could improve proliferation within a certain range. Meanwhile, it could significantly enhance the formation of ALP and calcium nodules in ASCs-T2DM, increase the expression of osteogenesis-related genes in vitro, and promote the healing of calvarial defects of T2DM rat in a concentration-dependent manner. 5-az intervention indicated that the reduction of the methylation level in Calca target fragment of ASCs-T2DM indeed escalated the gene expression, which may be related to DNMT1. Taken together, the environment of T2DM could upregulate the methylation level in the promoter region of Calca and then decrease the Calca expression. The coding product of Calca revealed a promoting role for osteogenic differentiation of ASCs-T2DM. This result provides an implication for us to understand the mechanism of the decreased osteogenic ability of ASCs-T2DM and improve its osteogenic capacity.

## 1. Introduction

Mesenchymal stem cells with the ability of multidirectional differentiation and self-renewal have been employed to repair and regenerate damaged tissues and organs, for instance, to speed up the healing process of bone and soft tissue trauma in diabetic patients [1]. Autologous stem cells have become optimal seed cells because of their clinical availability, long-term survival, and tolerance to transplantation without ethical issues [2]. Among them, bone marrow mesenchymal stem cells (BMSCs) and ASCs exhibit a promising prospect for regenerative therapies. Compared with BMSCs, ASCs are most widely used with simple clinical acquisition, little suffering, better proliferative potential and multidirectional differentiation, and more suitable for the standard culture environment [3, 4].

However, basic properties of ASCs derived from diabetic individuals have changed. T2DM is a typical metabolic disease characterized by hyperglycemia, abnormal insulin and cytokine levels, and oxidative stress. These factors contributed to the increase of fracture risks, the decline of fracture healing ability, and the formation of osteoporosis in diabetic patients [5]. Meanwhile, abnormal body status of T2DM affects the performance of the stem cells. Specifically, the osteogenic ability of ASCs and BMSCs from T2DM is weaker than that from healthy individuals, which cannot meet the demand for bone defect repair [6]. However, the gene sequence of diabetics did not vary before and after the disease. The difference between them may be due to the alterations of the expression of osteogenesis-related genes of stem cells caused by the change of the body environment.

Epigenetics is a bridge linking environmental and phenotypic changes, and DNA methylation is the most in-depth studied part, which refers to the addition of activated methyl to the 5-carbon end of cytosine (C) as methylcytosine [7]. It occurs in different regions of genes with varying effects on transcriptional regulation. When it appears in the promoter region, DNA methylation exerts an inhibitory effect on gene transcription. CpG (cytosine-phosphate-guanine) sites are enriched in the promoter region, which is called CpG islands [8]. In general, C in the CpG island of healthy people is nonmethylated. If the CpG islands are methylated, it will affect the activity of transcription factors or change the chromatin conformation to block access and binding of the transcription initiation complex and then result in gene silencing, ultimately affecting cell proliferation and differentiation [9]. It has been found that DNA methylation is important for diseases caused by the interaction of genetic and environmental factors and T2DM is one of them [9–11].

It is found that the environment of T2DM can change the level of DNA methylation at the whole genome level [12]. At the same time, diabetes is also prone to cause DNA methylation changes in peripheral tissue such as muscle and adipose [13]. In addition, diabetes is also closely related to the methylation of certain genes [14]. However, the underlying relationship between DNA methylation and stem cell from T2DM has not been studied too much, and the mechanism of DNA methylation and inferior osteogenesis of ASCs-T2DM patients remains elusive. In this study, we established T2DM models to extract ASCs for DNA methylation sequencing. Combined with sequencing data and literature analysis, we screened and verified the different genes that may affect the osteogenesis of ASCs in order to provide new ideas for related research.

## 2. Materials and Methods

### 2.1. Induction of T2DM Rat Models

SD rats (7-8weeks, 150-200 g, male) provided by the Experimental Animal Center (Accreditation No. SCXK 2014-002) were grown and processed in compliance with the Ethics Committee of the Fourth Military Medical University (Ethical Accreditation No. 2017kq-025). They were randomly divided into the T2DM group (*n* = 32) and the control group (*n* = 8). T2DM rats were induced by feeding with special feed (high fat and high sugar feed; basic feed, 69.5%; sucrose, 10%; egg yolk powder, 10%; cholesterol, 0.5%; fat, 10%) and intraperitoneal injection at the dose of 35 mg/kg STZ (Sigma, USA), while the control group received injection of citric acid buffer (Solarbio, China) at the same time combining with normal feed. A week later, blood from tail tips of rats was taken to measure the random glucose value and it was monitored weekly. Rats with random blood glucose above 16.7 mmol/L for four consecutive weeks were defined as successful models.

### 2.2. ASCs-C and ASCs-T2DM Isolation and Culture

ASCs were extracted by enzymatic digestion. First, rats of the T2DM and control groups were sacrificed and the adipose tissue in the groin was taken out, cut up, and digested with collagenase I (Gibco, USA). After then, the solution was mixed with the *α*-MEM complete medium (10% serum (Gibco, USA), 1% Penicillin-Streptomycin solution (HyClone, USA), and 89% basic medium (HyClone, USA)) and the mixture was filtered through 200 mesh screen. The filtrate was collected and centrifuged (1000 r/min, 5 min), then resuspended with complete medium. The cells were eventually plated in T75 flasks and incubated in CO_2_ incubators (Thermo, USA) until it spread more than 80% of the bottom to passage.

### 2.3. DNA Extraction and Methylation and Expression Analysis of Calca

DNA was extracted from ASCs-T2DM and ASCs-C, and purity and concentration were detected by a nucleic acid quantitative instrument (BioTek, USA). DNA samples with appropriate concentration and purity were selected for methylation sequencing. The final results were imported into visual IGV_2.3.97 to determine the methylation level and select differential gene combing with literature analysis. Meanwhile, RNA of ASCs-T2DM and ASCs-C was extracted, quantified, and reverse transcribed into complementary DNA (cDNA). qPCR was applied to detect the relative expression of Calca using a TB Green® Premix Ex Taq™ II kit (Takara, Japan). Setting *β*-actin as the endogenous reference, the relative expression of osteogenic gene was obtained by calculating the CT value. The primer sequences are listed in Table 1.

### 2.4. Morphological Observation and Cell Proliferation Analysis

ASCs-T2DM were inoculated in a 6-well plate at a density of 2.5 × 10^5^ and divided into four groups with three multiple wells. Then, medium containing different concentrations of CGRP (10^−7^ mol/L, 10^−8^ mol/L, 10^−9^ mol/L, and 0 mol/L) was added and changed until the cell confluence reached about 50%. Whereafter, they were fixed with 4% paraformaldehyde (Solaibio, China) and then membrane was ruptured with 0.5% Triton X-100 (Sigma, USA). After washing, 1% BSA (Solaibio, China) was added to block the antigen. The cells were covered with phalloidin staining (Sigma, USA) and incubated at room temperature for 30 minutes. Finally, they were dyed with DAPI (Solaibio, China) and observed under a fluorescence microscope (Olympus, Japan).

ASCs-T2DM were inoculated into 96-well plates at a density of 3000/well. Each group consisted of 3 multiple holes and 3 control holes. At the same time of each day, the mixture with rate 10 : 1 of CGRP (10^−7^ mol/L, 10^−8^ mol/L, 10^−9^ mol/L, and 0 mol/L) and CCK-8 (EnoGene, China) was added to the test group to incubate for 3 hours in CO_2_ incubators and then determine the absorbance value at 450 nm wavelength by a spectrophotometer (BioTek, USA). The average value was calculated, and the growth curve was drawn.

### 2.5. Osteogenetic Differentiation Induction and Staining

The ASCs-T2DM were inoculated into 6-well plates at a density of 2.5 × 10^5^/well. When the cell confluence reached 80%-90%, the osteogenic induction medium (10 mM *β*-glycerophosphate, 50 mg/mL ascorbic acid, and 10^−7^ M dexamethasone (MP, USA)) containing different concentrations of CGRP (10^−7^ mol/L, 10^−8^ mol/L, 10^−9^ mol/L, and 0 mol/L) was replaced. After 7 days and 28 days of induction, the cells were fixed with polyformaldehyde. BCIP/NBT staining solution and 0.1% alizarin red dye were prepared to determine the expression of alkaline phosphatase (ALP) and calcium nodule. Quantitative analysis of ALP was carried out by a microenzyme labeling method using the APK activity kit (Jiancheng, China). Hexadecylpyridine chloride eluent was added in with 1 mL/well to measure the absorbance value at 620 nm wavelength for semiquantitative analysis of alizarin red.

### 2.6. Real-Time Quantitative Polymerase Chain Reaction (qPCR)

ASCs-T2DM culture was performed in the same manner as in Section 2.5. On the 7th day of osteogenic induction, RNA was extracted from all the groups using RNAiso Plus (Takara, Japan), and 800 *μ*g total RNA was reverse transcribed to cDNA using a PrimeScript RT reagent kit (Takara, Japan). Osteogenic genes, including runt-related transcription factor 2 (*RUNX2*), alkaline phosphatase (*ALP*), collagen type I (*COL1*), osteocalcin (*OCN*), bone morphogenetic protein (*BMP*), were detected in the relative expression by qPCR. The primer sequences are listed in Table 1. The specific steps of this method were consistent with those in Section 2.3.

### 2.7. Creation of the Calvarial Defect Model and Transplantation of ASCs-Bone Powder Compound

The surgery was carried out under sterile conditions with anesthesia. Following disinfection and towel laying, an incision was made to expose the skull. Then, a defect with a diameter of 5 mm was formed by using a trephine and circular grinding drill under low speed and cooling saline between the coronal suture and the herringbone suture. Afterward, ASCs-T2DM suspension containing different concentrations of CGRP (10^−7^ mol/L, 10^−8^ mol/L, 10^−9^ mol/L, and 0 mol/L) was centrifuged and mixed with Bio-Oss® bone powder (Geistlich, Switzerland) and placed in an incubator for 30 minutes. Compounds were implanted into the skull defect site of T2DM rats. At 6 and 12 weeks after implantation, the specimens were obtained and fixed with 4% paraformaldehyde for CT scanning. Histological sections and H&E staining were performed after decalcification.

### 2.8. Target Fragment Validation of Calca

ASCs-T2DM cells were divided into the 5-az (a DNA methyltransferase inhibitor) group and the blank group (ASCs-T2DM). The blank group was cultured with normal medium, while the 5-az group was cultured with medium containing 5-az. DNA was extracted from both groups to detect the methylation level of target fragment in the promoter region of Calca by bisulfite amplicon sequencing. When the cells were treated for 72 hours, RNA was extracted from the two and qPCR was performed to detect the relative expression of DNMT1 (DNA methyltransferase 1) and Calca. The primer sequences are listed in Table 1. The specific steps of this method were consistent with those in Section 2.3.

### 2.9. Statistical Analyses

All values were reported as mean ± SD. Statistical analysis was performed using SPSS software. Unrelated *t*-test was used for comparison between the two groups. One-way ANOVA was used for comparison between different groups. The significance levels were set at 5% (at *P* < 0.05).

## 3. Results

### 3.1. Type 2 Diabetic Rat Models

After 1 week of modeling, the rats in the T2DM group showed obvious symptoms of constant hunger, polydipsia, polyuria and yellowish hair. Blood glucose of the T2DM rats was continuously maintained more than 16.7 mmol/L, while that of the rats in control group was lower than 8 mmol/L (Table 2). After 4 weeks, the rats in T2DM group showed obvious signs of emaciation, and their weight was significantly lower than that in the normal group. There were significant differences in blood glucose and body weight between the two groups (P < 0.01).

### 3.2. Methylation and Expression Level of Calca

**Figure 1:**
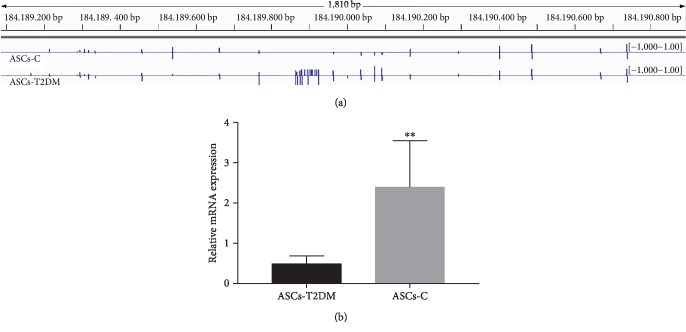
Methylation (a) and expression (b) level of Calca in ASCs-T2DM and ASCs-C. Notes: ^∗∗^*P* < 0.01. Abbreviation: mRNA: messenger RNA.

The promoter region of Calca is located in chr1:184188911-184190911, while its gene is in chr1:184184018-184188911. Visual analysis of IGV software showed that there were fewer methylation sites in the promoter region of the Calca in the ASCs-C than in the ASCs-T2DM (Figure 1(a)). The value of the methylation multiple of the two samples revealed that the methylation level in the promoter region of ASCs-T2DM was significantly increased compared with the control group, which confirmed that the methylation level in the promoter region of Calca could be upregulated in T2DM. Go analysis showed that the Calca was related to the regulation of osteogenesis, immunomodulation, angiogenesis, and other biological processes. At the same time, the expression of Calca decreased in ASCs-T2DM compared with ASCs-C (Figure 1(b)). The expression level of Calca was negatively correlated with the change of DNA methylation. In this regard, we conclude that T2DM improves the methylation level in promoter of Calca and then inhibits its expression in ASCs-T2DM, which may be related to the decreased osteogenic differentiation of ASCs in T2DM.

### 3.3. Morphological Observation and Cell Proliferation Analysis

**Figure 2:**
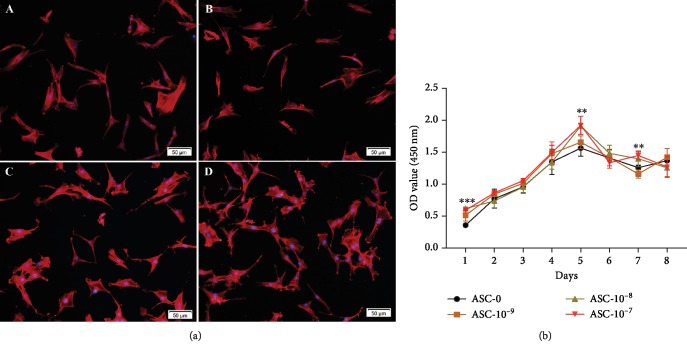
The morphology (a) and cell proliferation curve (b) of ASCs-T2DM with different concentrations of CGRP. Notes: (A) 0 mol/L, (B) 10^−9^ mol/L, (C) 10^−8^ mol/L, (D) 10^−7^ mol/L. Scale bar = 50 *μ*m; ^∗^*P* < 0.05; ^∗∗^*P* < 0.01; ^∗∗∗^*P* < 0.001.

Four groups of ASCs-T2DM were added with different concentrations of CGRP. The results showed that the cells in each group were spindle-shaped, polygonal, or star-shaped. There was no significant difference in cell morphology between different concentrations of CGRP. The results indicated that the addition of Calca in vitro had no significant effect on cell morphology (Figure 2(a)).

The significant S-shaped growth curves were formed in four groups, and cell proliferation reached a peak on the 5th day. Different concentrations of CGRP could affect the proliferation of ASCs-T2DM. The medium of 10^−7^-10^−8^ mol/L could significantly increase the proliferation capacity of ASCs-T2DM. On the 5th and 7th days, there were significant statistical differences among the groups (Figure 2(b)).

### 3.4. Osteogenesis Staining

**Figure 3:**
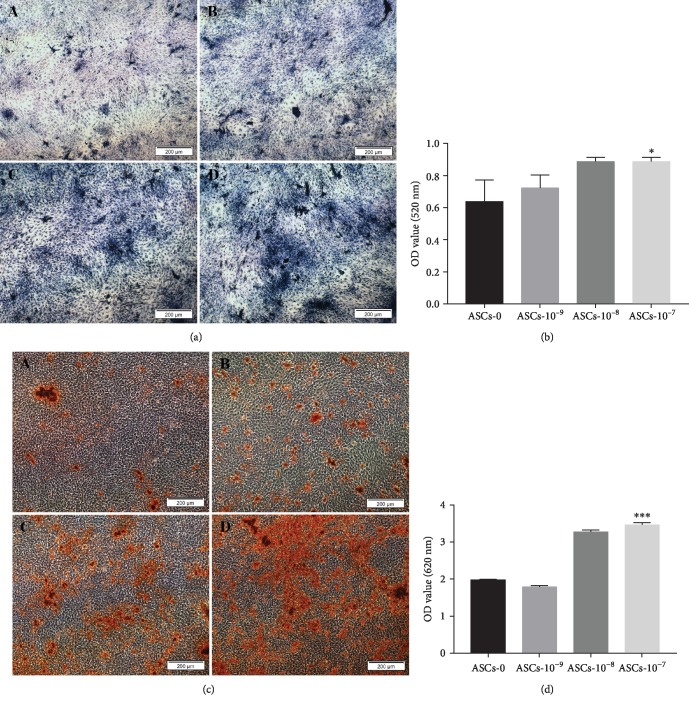
Osteogenic staining of ASCs-T2DM with different concentrations of CGRP and quantitative comparison. (a) BCIP/NBT staining for ALP after 7-day osteogenic differentiation. (b) Quantitative comparison of BCIP/NBT staining using AKP kits. (c) Alizarin red staining for mineralized nodules after 28-day osteogenic differentiation. (d) Quantitative comparison of alizarin red staining using a spectrophotometer at 620 nm. Notes: (A) 0 mol/L, (B) 10^−9^ mol/L, (C) 10^−8^ mol/L, and (D) 10^−7^ mol/L. Scale bar = 200 *μ*m; ^∗^*P* < 0.05; ^∗∗∗^*P* < 0.001.

After 7 days of osteogenic induction with different concentrations of CGRP, ALP staining showed that ASCs-T2DM in all groups could be blue-purple stained. With the increase of CGRP concentration, the color of cells deepened gradually and the area of color development expanded (Figure 3(a)). At the same time, quantitative studies indicated that the expression of ALP elevated with the increase of CGRP concentration (Figure 3(b)). Statistical analysis showed that the difference was statistically significant (*P* < 0.05). After 28 days, alizarin red staining demonstrated that mineralized nodules were formed in different groups and the number and volume of nodules increased with the increase of CGRP concentration. Most nodules were formed in the 10^−7^ mol/L group, and even some of them were connected into sheets (Figure 3(c)). Quantitative analysis exhibited that CGRP could promote the formation of calcium nodules in osteogenic differentiation of ASCs-T2DM in a concentration-dependent manner (Figure 3(d)). Statistical analysis showed that the difference was statistically significant (*P* < 0.001).

### 3.5. Osteogenesis-Related Gene Expressions

**Figure 4:**
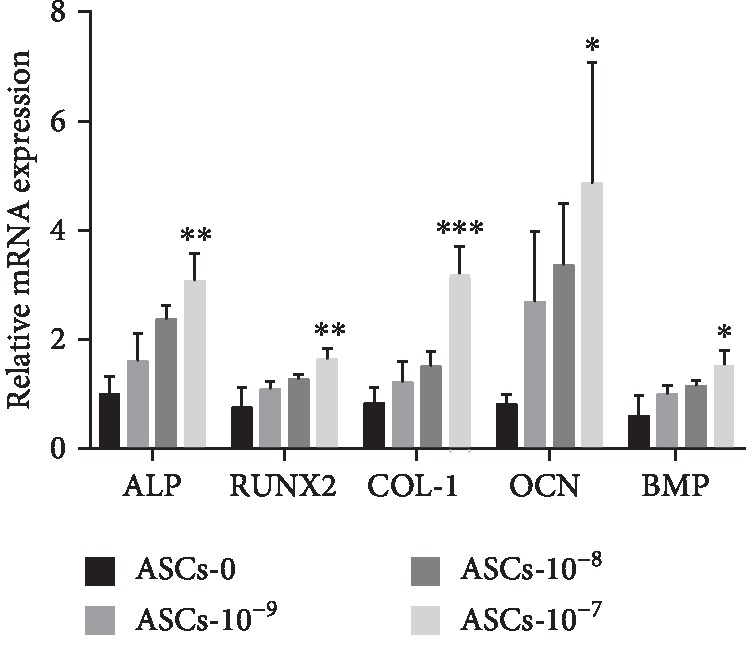
Expression of osteoblast-related genes of ASCs-T2DM with different concentrations of CGRP. Notes: ^∗^*P* < 0.05; ^∗∗^*P* < 0.01; ^∗∗∗^*P* < 0.001. Abbreviations: *ALP*: alkaline phosphatase; *RUNX2*: runt-related transcription factor 2; *COL-1*: collagen type I; *OCN*: osteocalcin; *BMP*: bone morphogenetic protein; mRNA: messenger RNA.

The relative expression levels of *ALP*, *RUNX2*, *COL-I*, *OCN*, and *BMP* were analyzed by qPCR. On the whole, the expression of osteogenesis-related genes increased after intervention with different concentrations of CGRP and exhibited a concentration-dependent trend. When the 10^−7^ mol/L CGRP was added, the effect of promoting osteogenesis was strongest. Among them, the *COL-1* gene had the most significant difference in different concentration groups, while the *OCN* gene, as an important gene related to osteoblasts, had the most expression in each group. By contrast, the expression of *BMP* in each group differed slightly (Figure 4).

### 3.6. Micro-CT and Histological Analysis

**Figure 5:**
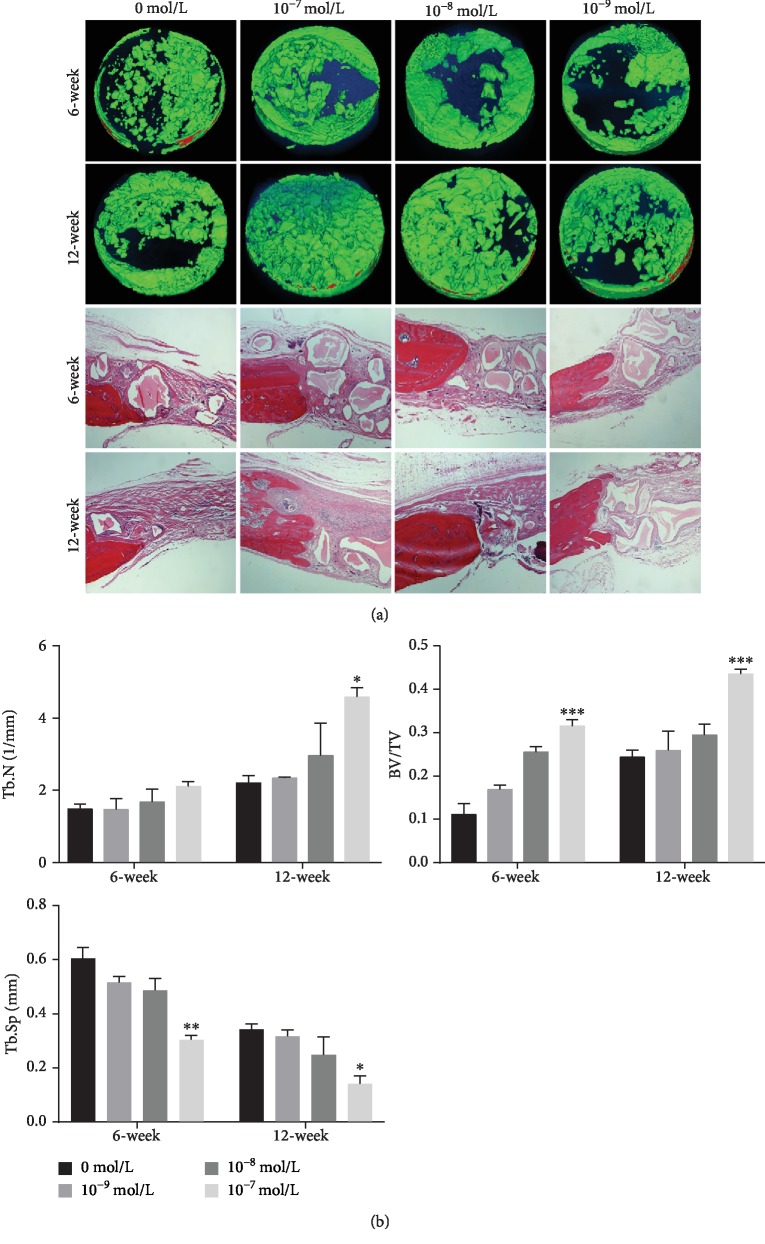
Micro-CT scanning, H&E staining, and quantitative analysis at 6 and 12 weeks. (a) Micro-CT scanning of bone defect models with ASCs-Bioss compound with different concentrations of CGRP at 6-week and 12-week postimplantation. Histological analysis by H&E staining after 6 and 12-week healing of bone defect models with ASCs-bone powder compound. (b) Quantitative analysis of regenerated bone was evaluated in Tb.N, BV/TV, and Tb.Sp. Notes: H&E staining (stereo microscope, 100x); ^∗^*P* < 0.05; ^∗∗^*P* < 0.01; ^∗∗∗^*P* < 0.001. Abbreviations: CT: computed tomography; Tb.N: trabecular number; BV/TV: bone volume to tissue volume; Tb.Sp: trabecular separation.

The area with diameter of 5 mm and thickness of 1 mm in the skull center was defined as region of interest (ROI) area, and quantitative analysis was carried out according to the CT value. The new bone is in green with the 700-2000 CT value, and the bone powder is in red when the value is more than 2000. The results demonstrated the newly formed bone in the edge at 6 weeks in all groups. However, there was still a low-density shadow in the central area, that is, obvious bone defect, in which the defect was larger in the low-concentration CGRP group and control group. At 12 weeks, the defect area in each group was reduced and it tended to heal with 10^−7^ mol/L CGRP. However, there was a large range of the nonhealing area in the 10^−9^ mol/L and control groups (Figure 5(a)). The quantitative analysis of ROI showed that the trabecular number (Tb.N) improved with the increase of concentration at 6 weeks and this trend continued over time. The result of new bone volume/tissue volume (BV/TV) was consistent with the trend of Tb.N. Contrasting results were seen in trabecular separation (Tb.Sp). As time goes by, it exhibited a decrease and possessed a negative correlation with the intervention concentration of CGRP (Figure 5(b)). H&E results revealed that bone tissue like finger in the marginal area protruded into the central defect area at 6 weeks, but a large amount of bone powder was still surrounded by fibrous tissue. At 12 weeks, the amount and volume of bone powder decreased, the bone growth in the defect was obvious, and the loose fiber bundle was gradually dense to form dense tissue. Bone precursor structure and more finger-like tissue were noted at the edge of the defect in the10^−7^ mol/L group whereas a large number of fascicular or layered fibrous tissue were reported in the 0 mol/L group (Figure 5(a)).

### 3.7. Target Fragment Validation of Calca

**Figure 6:**
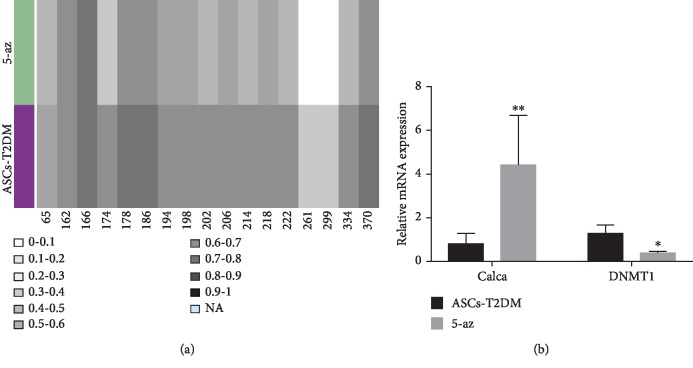
Methylation level and gene expression analysis. (a) Methylation level of target fragment of Calca in different sites. Abscissa: CG site, the methylation level of which is represented by different gray blocks. (b) Expression analysis of Calca and DNMT1 in the blank and 5-az groups. Notes: ^∗^*P* < 0.05; ^∗∗^*P* < 0.01.

Comparing the methylation in target fragments of Calca at different sites between two groups, it was found that the methylation level at most sites in the 5-az group was lower than that in the blank group, which suggested that 5-az exerts an impaired effect on the Calca methylation process in target fragments (Figure 6(a)). The expression of Calca and DNMT1 genes was detected by qPCR, which showed that the Calca expression increased significantly while the expression of DNMT1 decreased with the 5-az intervention (Figure 6(b)).

## 4. Discussion

In recent years, mesenchymal stem cells with advantages of self-renewal and multidirectional differentiation are widely used to improve the osteogenesis of diabetic individuals, which have been confirmed by many studies [15, 16]. Autologous stem cells have become promising cells due to long-term transplant survival and tolerance and clinical availability. However, BMSCs-T2DM are accompanied by impaired proliferation, early proliferative aging, strict culture conditions, and greater pain [6]. By comparison, ASCs-T2DM is a superior choice with simple clinical acquisition, more stable characteristics, and adaptability to standard culture environment [4]. In addition, the number of ASCs from diabetic individuals does not decrease and the growth curve is similar to that of the control group [17]. However, it is undeniable that ASCs-T2DM have less osteogenic ability than those from healthy individuals. However, the gene sequence has not changed before and after the disease in the same patient. All of them are thought to be associated with changes of osteoblast-related gene expression acting via epigenetic modifications.

Epigenetics mainly involves the interaction between external environment and internal gene [18]. DNA methylation is the most widely studied part which mainly regulates gene expression at a transcriptional level with nonchanging DNA sequence and stable inheritance [19]. Specifically, activated methyl from *S*-adenosylmethionine is added to the 5-carbon end of cytosine to modify it as 5-methylcytosine [20, 21]. Moreover, their level varies according to environmental factors. DNA methylation could be divided into de novo methylation and maintenance methylation. DNMT1 is responsible for the maintenance of DNA methylation in mammals, whose effect could be inhibited by inhibitors. DNMT inhibitors are mostly nucleoside analogs of cytosine, which are incorporated into new synthetic chains when DNA is in process of semiconservative replication and competently bind to DNMTs to inactivate them and block DNA methylation, such as 5-azacytidine [22]. DNA methylation occurring in different regions possesses different effects on gene expression. Generally, methylation in the promoter region could enhance transcriptional inhibition, but the effect in gene region has not been conclusive [23]. As a metabolic disease interacting with environmental and genetic risks, T2DM was found to be associated with DNA methylation level changes at the whole genome [24] and CpG-labeled DNA methylation has also been found as a biomarker of metabolic syndrome [25]. At the same time, diabetes mellitus could lead to the change of the methylation level in specific tissues, such as adipose tissue, while the related differential genes are mainly related to cell cycle regulation and damage response [26].

In order to explore the underlying mechanism of T2DM affecting ASCs, we established the T2DM rat model to find out the different genes affecting the osteogenesis of ASCs-T2DM. Considering the instability before and after modeling using single individual, we chose to study the methylation difference between ASCs-T2DM and ASCs-C in the promoter region. Then, we extend this effect (T2DM status) to ASCs, so as to clarify the relationship between the T2DM environment and ASCs osteogenic ability. We extracted DNA from ASCs-T2DM and ASCs-C and sequenced their genomic DNA methylation. According to the GO and visual software analysis, we selected differential genes related to osteogenic differentiation or osteogenesis in the promoter region and excluded the false-positive results. Combined with the qPCR, candidate genes with a negative correlation between the DNA methylation and gene expression level were chosen. Ultimately, Calca, with a higher DNA methylation and a lower expression level in ASCs-T2DM than ASCs-C, was set as an object of study.

Calca, as an osteogenesis-related gene, could encode two peptides: calcitonin (CT) and *α*-calcitonin gene-related peptide (*α*-CGRP) [27]. However, there was no significant sequence homology between the two after processing from inactive precursors [28]. CT exists in renal epithelial cells, neurons of the central nervous system, placental cells, or lymphocytes, mainly regulating bone resorption (inhibiting bone resorption) [29]. *α*-CGRP mainly distributes in the central and peripheral nervous systems, as well as in some endocrine tissues, which mainly participate in bone synthesis, pain response, mineral metabolism, and other processes [30]. Studies involved with *α*-CGRP initially focused on the nervous system and the cardiovascular system [31]. Recent studies found that it could also affect osteogenic differentiation of mesenchymal stem cells to regulate bone repair and reconstruction. Huang et al. used CGRP to promote the osteogenic differentiation of ASCs with calcium alginate gel and results revealed that ALP and the calcified nodules staining increased significantly in the CGRP induction group than the control group, which confirmed that CGRP could promote the differentiation of ASCs into osteoblasts [32]. Zhou et al. noted that CGRP enhanced the expression of osteoblastic marker genes in the osteoblastic differentiation of BMSCs throughout the Wnt signaling pathway [33]. Besides, CGRP can not only promote the proliferation and activity of osteoblasts but also inhibit its apoptosis [34]. Simultaneously, it can also inhibit the activity of osteoclasts, reduce the proliferation and differentiation of osteoclast precursors, and retard bone resorption [35]. *α*-CGRP not only acts as a regulator of bone metabolism but also participates in angiogenesis during bone repair, thus promoting the healing of bone or other tissue traumas [28].

Subsequently, in order to verify the effect of Calca on osteogenic differentiation of ASCs-T2DM, we select CGRP, a vitro analog of the transcription product of Calca, to intervene ASCs-T2DM. Results showed that CGRP could not affect the morphology of ASCs-T2DM but could enhance its proliferation in a certain extent. In addition, CGRP can significantly escalate the expression of osteogenesis-related genes and the formation of ALP and calcium nodules in a concentration-dependent manner. Meanwhile, we established the T2DM rat model of skull defect to verify the effect of CGRP on the osteogenesis of ASCs-T2DM in vivo. ASCs-T2DM were implanted into the skull defect after mixed with bone powder, and the healing status was monitored and observed at different times. The results demonstrated that there was a small amount of bone formation on the edge of the defect in the early stage. However, a large amount of bone powder still existed and were surrounded by fibrous tissue. At 12 weeks, the amount and volume of bone powder decreased and the loose fiber bundle was gradually dense to form osteoid precursor tissue. Among them, 10^−7^ and 10^−8^ mol/L groups showed a strong promotion to bone formation. In order to further verify the relationship between DNA methylation in the target fragment and Calca expression of ASCs-T2DM and figure out specific mechanisms, we selected 5-az to intervene ASCs-T2DM, which could specifically inhibit DNA methylation by acting on DNMT1. The results indicated that 5-az could downregulate the DNA methylation level of the target fragment in the Calca promoter region of ASCs-T2DM, which may be regulated by reducing the expression of DNMT1, thereby elevating the expression of Calca.

We selected two groups of cells for comparative analysis and found that the influence of the T2DM environment on ASC osteogenesis may be related to the decrease of expression caused by the increase of the methylation level of Calca. It is further speculated that the environment will change the methylation level of osteogenesis-related genes in ASCs and then affect their expression for the patient with T2DM. However, it is undeniable that this experimental design is based on the group data, not the direct analysis before and after a person's illness. In addition, the final validation of the experiment did not directly modify the target fragment to verify the osteogenic effect. So, our next work is to verify the different genes before and after the individual's disease and modify the target fragment by gene editing. However, this research bores out that the DNA methylation status in the promoter has a strong inverse correlation with gene expression and gives us idea that the interaction may be a potential therapeutic target in the future.

## 5. Conclusions

The T2DM environment could upregulate the DNA methylation level of the target fragment in the Calca promoter, thus reducing the expression of Calca in ASCs-T2DM. The coding product of Calca exerts the stimulative effect on the osteogenesis of ASCs-T2DM in vitro and in vivo. It is suggested that we can improve the osteogenesis of ASCs-T2DM by changing the DNA methylation level of genes related to osteogenesis.

## Figures and Tables

**Table 1:** Primer sequences for qPCR.

Gene	Forward primer sequence (5′-3′)	Reverse primer sequence (5′-3′)
*β-Actin*	TGGCACCCAGCACAATGAA	CTAAGTCATAGTCCGCCTAGAAGCA
*RUNX2*	CCATAACGGTCTTCACAAATCCT	TCTGTCTGTGCCTTCTTGGTTC
*ALP*	CCTTGTAGCCAGGCCCATTG	GGACCATTCCCACGTCTTCAC
*COL-1*	GCCTCCCAGAACATCACCTA	GCAGGGACTTCTTGAGGTTG
*OCN*	CCCAGGCGCTACCTGTATCAA	GGTCAGCCAACTCGTCACAGTC
*BMP*	CAACACCGTGCTCAGCTTC C	TTCCCACTCATTTCTGAAAGTTCC
*Calca*	AGTCATCGCTCACCAGGGA	GGCTGCTTTCCAAGGTTGAC
*DNMT1*	TATTGCAGTCGCGGTCACTT	CTGATTGATTGGCCCCAGGT

Abbreviations: *RUNX2*: runt-related transcription factor 2; *ALP*: alkaline phosphatase; *COL-1*: collagen type I; *OCN*: osteocalcin; *BMP*: bone morphogenetic protein; *Calca*: calcitonin-related polypeptide; *DNMT1*: DNA methyltransferase 1.

**Table 2:** Comparison of blood glucose values between T2DM and Control rats (mmol/L, $x\pm \bar{s}$).

Group	Before	1 W	2 W	3 W	4 W
T2DM	6.80 ± 0.398	28.84 ± 1.502	27.77 ± 1.273	27.86 ± 1.289	27.73 ± 1.460
Control	6.81 ± 0.159	6.71 ± 0.336	6.68 ± 0.354	6.90 ± 0.071	6.78 ± 0.424

Abbreviations: T2DM: type2 diabetes mellitus; W: week.

## Data Availability

The datasets used and/or analysed during the current study are available from the corresponding author on reasonable request.

## Abbreviations

*α*-CGRP: *α*-Calcitonin gene-related peptide
ALP: Alkaline phosphatase
ASCs: Adipose-derived stem cells
BMP: Bone morphogenetic protein
BMSC: Bone marrow mesenchymal stem cell
BSA: Bovine serum albumin
BV/TV: Bone volume to tissue volume
C: Cytosine
Calca: Calcitonin-related polypeptide
CCK-8: Cell counting kit-8
cDNA: Complementary DNA
COL- I: Collagen type I
CpG: Cytosine-phosphate-guanine
CT: Computed tomography
DAPI: Diamidino phenylindole
DNMT1: DNA methyltransferase 1
OCN: Osteocalcin
PBS: Phosphate buffer solution
qPCR: Real-time quantitative polymerase chain reaction
RUNX2: Runt-related transcription factor 2
STZ: Streptozocin
T2DM: Type 2 diabetes mellitus
Tb.N: Trabecular number
Tb.Sp: Trabecular separation
5-az: 5-Azacytidine.
